# Fabrication, Characterization and Toxicity Evaluation of Chemically Cross linked Polymeric Network for Sustained Delivery of Metoprolol Tartrate

**DOI:** 10.1080/15685551.2021.2003995

**Published:** 2021-12-09

**Authors:** Sumaira, Ume Ruqia Tulain, Alia Erum, Muhammad Ajaz Hussain, Sidra, Nadia Shamshad Malik, Ayesha Rashid, Rizwana Kausar, Nitasha Gohar, Nariman Shahid, Mahwish Siddiqui

**Affiliations:** a Faculty of Pharmacy, University of Sargodha, Sargodha, Pakistan; b Institute of Chemistry, University of Sargodha, Sargodha, Pakistan; c Faculty of Pharmacy, Capital University of Science and Technology, Islamabad, Pakistan; d Department of Pharmacy, Women University Multan, Multan, Pakistan; e Ilm College of Pharmaceutical Sciences, Sargodha, Pakistan

**Keywords:** Natural mucilages, sustained delivery, toxicity evaluation, polymeric network, chemically crosslinked

## Abstract

Natural mucilages are auspicious biodegradable polymeric materials. The aim of the present research work was to elucidate the characteristics of quince mucilage-based polymeric network for sustained delivery of metprolol tartrate and its toxicity evaluation. Mucilage was extracted by hot water extraction, and characterization of quince mucilage was accomplished by using Fourier transform infrared (FTIR) spectroscopy. Different batches of quince mucilage polymeric network were prepared by free radical polymerization by utilizing varying ratios of quince mucilage, acrylamide and crosslinker. Degree of swelling depends on concentration of mucilage, monomer and also on crosslinking density of polymeric network. FTIR illustrates proficient grafting, and morphological (scanning electron microscopy) analysis signified porous design. Hence, quince mucilage-based design was encouraging for sustained delivery of metprolol tartrate and acute toxicity evaluation proved that mucilage-based network was safe for oral drug delivery system.

## Introduction

Hydrogels are 3D hydrophilic polymeric networks capable of absorbing huge volume of water and biological fluids. Their swelling and deswelling, elastic and tissue imitating properties demonstrated them as potential entrant for drug delivery 1. Hydrogels can be diligently used as an intelligent drug delivery system 2.

Variable physical stability and crystalline regions restrict drug-poor absorption potential. However, hydrogels owe to the presence of extraordinarily high levels of absorption. In hydrogel synthesis, biopolymers are gaining attention as renewable materials with biocompatible and environmentally sustainable characteristics, and natural sources of hydrophilicity and biocompatibility are the main characteristics of a biopolymer at the root [3].

The mucilage exhibits some encouraging properties, such as a powerful swelling and slippery presence, possibly because of carbohydrate structure hydration, which has been observed to increase the lubrication of certain other structures dependent on water [4]. Quince mucilage was extracted by hot water extraction from the quince mucilage seeds with a wide variety of applications in the pharmaceutical sector. Thanks to their high water content, hydrogels appear more like natural soft tissue than any other form of polymeric biomaterial. Much of the recent interest in quince mucilage however is based on the polysaccharides it contains that are water-soluble. There are many studies on the isolation and purification of the unique glucuronoxylan from quince seed mucilage (QCM). QCM contains water-soluble polysaccharides, composed of D-xylose, L-arabinose and aldobiouronic acid [5,6].

Metprolol tartrate (MT) has cardio-selective competitive beta-1 adrenergic receptor antagonist properties and is a BCS type 1 antihypertensive medication. MT is completely consumed after oral administration, but only 50% become bioavailable due to the first pass effect and it also has a shorter half-life. It is thus known as an appropriate candidate for the production of extended release formulations [7].

Acrylamide is used as a monomer, and methylene bisacrylamide is used as a crosslinker, which persuades swelling, medication loading and release. Physically, polymer and monomer are intertwined, hydrogen bound, bonded or covalently by van der Waals forces. A molecular object that can create a crosslink point by connecting two or more polymer chains and forming a network structure is called as a crosslinking agent or a crosslinker [8,9].

The lack of protection and regulatory requirements for excipients initiated the creation of guidance redefining excipients as products that exhibit little or rather no pharmacological action, and the explanation for this is that excipient safety evaluation is less difficult and less detailed relative to active agents [10].

In this work, for controlled delivery of readily metabolized metoprolol tartrate, hydrogels with natural polymeric extract of QSM with acrylamide were prepared. By free radical polymerization, different batches of QSM were formulated by using multiple polymer, monomer and crosslinker ratios. In order to assess the safety profile of quince mucilage, a detailed in vitro and in vivo toxicity covering physical, biochemical and histopathological assessment was performed.

## Materials and methods

Quince seeds were secured from the local market of Sargodha. Methylene bis-acrylamide (MBA) and Acrylamide (AA) were obtained from Merck, Germany. Potassium per sulphate (KPS) was purchased from Sigma-Aldrich. The College of Pharmacy, University of Sargodha, granted sodium hydroxide (NaOH), hydrochloric acid (HCl), potassium dihydrogen phosphate (K2H_2_PO_4_) and ethanol.

### Extraction of QSM

Hot water extraction method was acquired to extract QSM. Debris collection was accompanied by sorting of 200 g of plants. In 800 mL of water, quince seeds were soaked for 8 h before heating for 1–2 h at 60**°**C. Through using cotton fabric, the extruded mucilage was removed and washed three times with *n*-hexane to eliminate lipophilic compounds. It was then dried in a 45**^o^**C hot air oven. With pestle and mortar, dried mucilage was ground and sieved using 80 mesh sieves [11].

### Preparation of quince mucilage-based polymeric network

**Figure 1.**
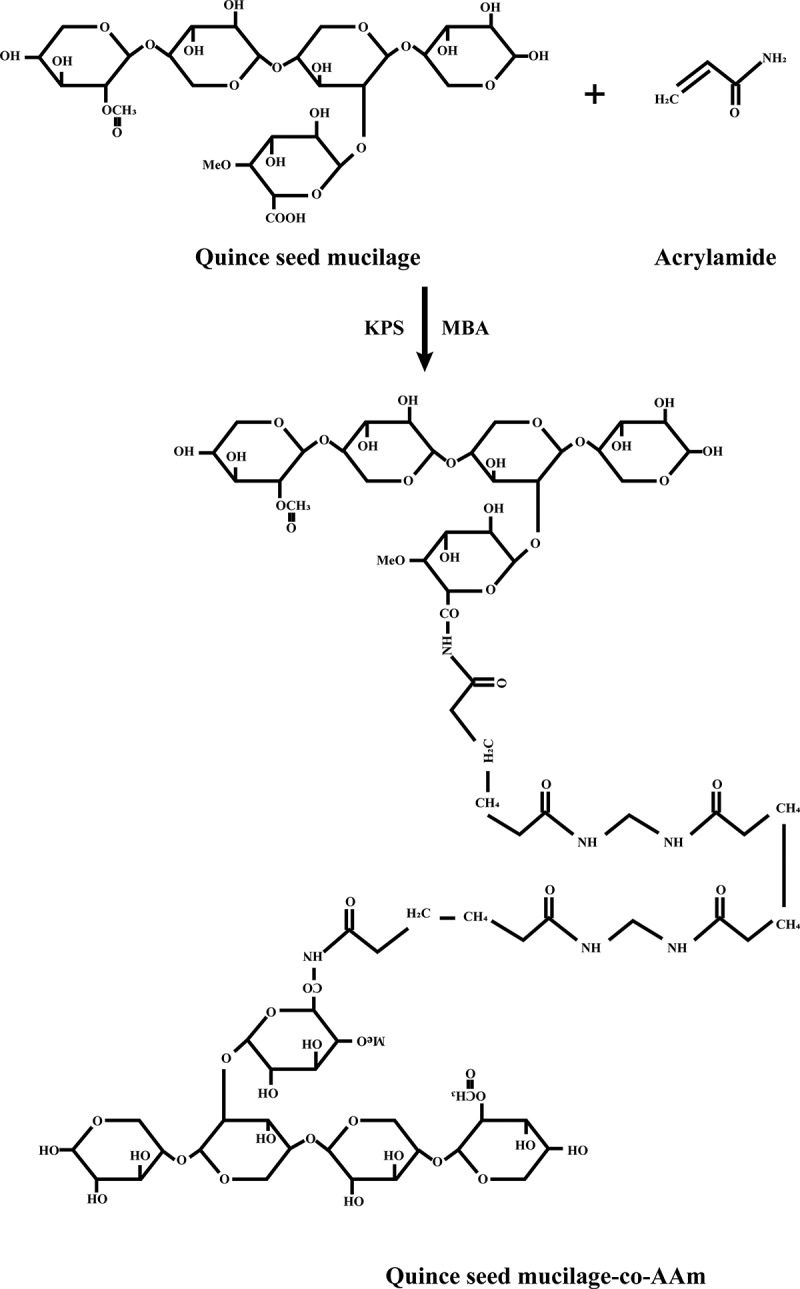
Bonding of QSM-co-AAm polymeric network

The hydrogels of QSM were prepared using the free radical polymerization process. As shown in Table 1, various formulations were prepared using differing ratios of acrylamide monomers, crosslinker and different polymer ratios. Dissolving quince mucilage in distilled water at 70**°**C on a hot plate by continuous stirring with the magnetic stirrer was prepared for the formulation of hydrogel polymer solution. KPS as the initiator was slowly applied under continuous stirring in polymer solution; the mixture was cooled to room temperature. With the aid of a magnetic stirrer, the crosslinker was then dissolved in monomer. This solution was then poured into the polymer solution at room temperature with constant stirring. The final weight of this hydrogel preparation was made up to 100 g with distilled water and stirred for 2 min. Nitrogen (as free radical scavenger) was bubbled for 15–20 min to remove dissolved oxygen to avoid inhibition of polymerization reaction. Polymerization was carried out in water bath at 55**^o^**C for 1 h, then 65**°**C for another hour and finally 80**°**C before the reaction was completed. The creation of bubbles has been prevented by incremental temperature changes. Hydrogels were extracted from the water bath after the reaction had been completed and cooled to room temperature. With the assistance of a sharp cutter, the formulated hydrogel was cut into 0.5-cm-thick discs. These discs were washed for 24 h with more than 30% ethanol:water solution in distilled water to eliminate unreacted monomer and reagent excess. After 24 h, the ethanol solution was altered for another 24 h with a fresh solution. Hydrogel discs were oven-dried at 50**°**C after full washing until the constant weight of hydrogels was collected. For further assessment, dried discs were stored [12]. The proposed bonding of QSM-co-AAm polymeric network is presented in Figure 1.

**Table 1.** Composition of 100 g QSM-co-acrylamide hydrogels

**Formulation code**	**Polymer** **quince mucilage (g)**	**Crosslinker** **MBA (% mole ratio of monomer)**	**Initiator** **KPS (% mole ratio of monomer)**	**Acrylamide** **(g)**
M-3	1	0.02	0.02	10
M-4	1	0.02	0.02	17.5
C-3	1	0.03	0.02	15
C-4	1	0.04	0.02	15
P-3	0.5	0.02	0.02	15
P-4	1	0.02	0.02	15

### Swelling studies

Dry discs were weighted and soaked in pH 1.2 and pH 7.4 buffer solutions in USP apparatus. After regular time intervals, samples were taken from buffer solutions and excess water was extracted by blotting using laboratory tissue prior to weighing. After dynamic swelling was achieved, samples were held in the same solutions and used for equilibrium swelling [13].

The dynamic swelling ratio of different formulations was obtained by the following equation:

(Equation 1) $$Q=\frac{W_{h}}{W_{d}}$$

where *W*_d_ depicts weight of the dried hydrogel disc, ‘*Q*’ represents dynamic swelling, and *W*_h_ show swollen gel’s weight at time *t* [13].

The equivalent weight of QSM-co-AAm was used for the determination of percent equilibrium swelling (%ES) for formulation by using the following equation:

(Equation 2) $$\%ES=\frac{Meq-M0}{Meq}*100\;$$

where *M*_0_ and *M*_eq_ show the mass of dried and swollen gel discs at equilibrium, respectively.

### Drug loading

The absorption process was used to load the QSM-co-AAm polymeric network. Drug solution in the pH 7.4 phosphate buffer was made. One disc was submerged in 100 mL of drug solution before the swelling balance was attained for all formulations. After the achievement of swelling equilibrium, discs were separated from the solution; by washing with purified water, excess surface drug was removed. It was first allowed to dry at room temperature and then dried at 50**°**C before the drying equilibrium was reached [14].

Loaded drug in QSM-co-AAm polymeric network discs was measured by the following formula:

(Equation 3) $$Totaldrugloaded=W_{l}--W_{u}$$

where *W*_l_ and *W*_u_ is weight of the dried drug loaded and unloaded discs, respectively.

### Fourier transform infrared (FTIR) analysis

To crush samples, pestles and mortar were used. The crushed material was blended in 1:100 proportions with potassium bromide (Merck IR spectroscopy grade) and dried at 40**°**C and then compressed into a semi-transparent 12 mm disc with a pressure of 60 kN (pressure gauge, Shimadzu) for 2 min. The FTIR spectrum was recorded using an FTIR spectrometer over a wavelength range of 4000–500 cm^−1^ (FTIR 8400S, Shimadzu) [15].

### Scanning electron microscopy (SEM)

SEM was conducted to observe the surface morphology and porosity of quince hydrogels using the scanning electron microscope (Hitachi, S 3000H, Japan). The samples were placed on an aluminium mount and gold palladium coated with sputter coater. For scanning, an accelerating voltage of 10 kV was used with a working distance of 10–25 mm [16].

### In vitro drug release measurement

In vitro drug release analysis was conducted on hydrogel discs filled with metoprolol tartrate according to the U.S. Pharmacopoeia requirements using USP apparatus II. Also, 900 mL of relevant dissolution media were used, i.e. 0.2 M HCl (pH 1.2) and pH 7.4 0.2 M phosphate buffers, and stirred at 50 rpm at 37**°**C. At 0, 0.5, 1, 2, 4, 6, 8, 10, 14, 18, and 24 h after filtering, 5 mL of aliquot was removed. To preserve volume at each interval, 5 mL of fresh medium was added. Samples were diluted with the required buffer and analysed at 222 nm using a cumulative drug release analysis UV-spectrophotometer (Shimadzu, Japan) in triplicate and recorded as a mean drug release study [17].

### Percentage drug release

Percent drug release of qQSM-co-AAm hydrogel was determined using the following equation:

(Equation4) $$\%\;drug\;release=\frac{F1}{F\;load}*100$$

where the quantity of metoprolol tartrate released at time *t* is shown by *F*_1,_ and the quantity of metoprolol released at time *t* is represented by *F*_load_ in hydrogel [17].

### Evaluation of release kinetics

QSM-co-Aam hydrogel drug release analysis zero-order, first-order, Higuchi and Korsmeyer–Peppas models were used to get an insight into the solute release mechanism [17].

### Acute toxicity in mice

Acute toxicity testing of the hydrogels was performed in Swiss albino mice (28- 34g). Animals were kept in the animal house of the University of Sargodha in clean cages in light/dark cycles of 12 h. Animals were fed with standard laboratory diet and water. The animal laboratory care guidelines (Canadian Council on Animal Care, 1993) were strictly followed.

### Acute toxicity in rabbits

The hydrogels were also tested in Albino rabbits (1–1.5 kg) of either sex. Rabbits were kept in the animal house of the University of Sargodha in clean cages and under 12 h light/dark cycle. Animals were fed with standard diet and water. Rabbits were divided into two groups (*n* = 3). Group 1 was served as control. Group 2 was given orally 2 g/kg body weight.

### Physical observation and mortality

Animals were observed on a daily basis for any sign of ill health, the reaction towards treatment, and changes in eyes, mucous membranes, skin, fur, tremors, salivation, sleep, behaviour pattern, faeces activity, coma and death for a period of 14 days.

### Body weight, food and water consumption

Body weights of the animals were checked before treatment and then on 1st, 7th and 14th days of the treatment. Consumption of water and food was also recorded on the same days and measured with that of the control group.

### Biochemical and haematological assay

After 14 days of treatment, the blood from rats was collected via cardiac puncture, under anaesthesia, in different sampling vials for haematological and various biochemical analyses. Biochemical analyses included liver function tests (LFTs), lipid profiles, haemoglobin (Hb) and urea. For rabbits, the blood was collected from the jugular vein and same biochemical evaluation was performed.

### Statistical analysis

The results were statistically analysed using one-way. *p* < 0.05 was considered as significant. The results were performed in triplicate and presented as mean ± SD.

## Results

**Figure 2.**
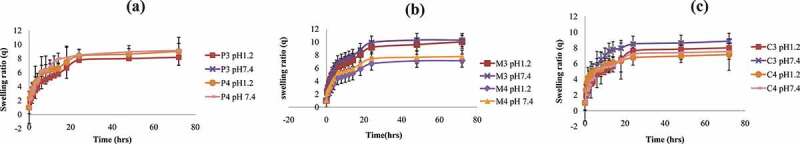
Swelling action of various hydrogel formulations

**Figure 3.**
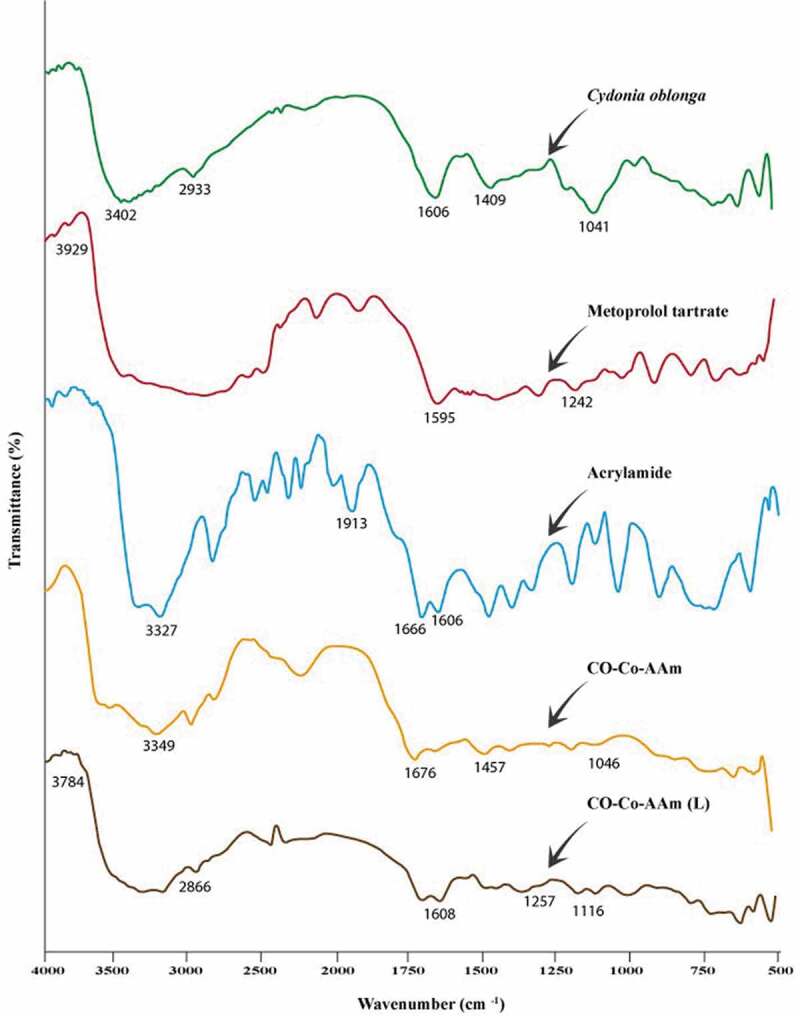
FTIR of various hydrogel formulations

**Figure 4.**
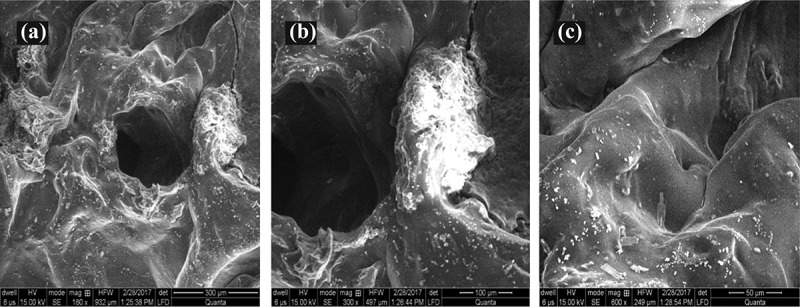
SEM of various hydrogel formulations

**Figure 5.**
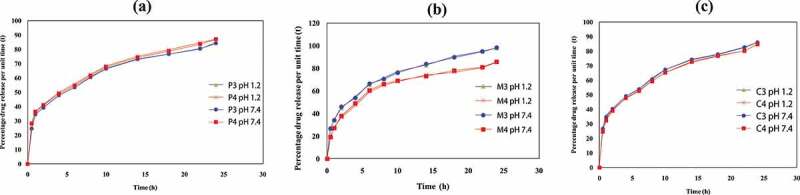
Percentage drug release of various hydrogel formulations

Results are shown in Figures 1–5 and Tables 2-5.

**Table 2.** Application of drug release models on dissolution data of QSM-co-acrylamide hydrogels

Formulation code	Zero-order model		First-order model		Higuchi model		Korsmeyer–Peppas model			Hixson–Crowell model	
	*R*^2^	*K*_0_	*R*^2^	*K*_1_	*R*^2^	kH	*R*^2^	kKP	*n*	*R*^2^	kHC
M3	0.253	5.170	0.895	0.188	0.902	22.359	0.997	35.519	0.323	0.839	0.050
M4	0.269	4.527	0.850	0.130	0.899	19.618	0.983	30.452	0.332	0.754	0.035
C3	0.153	4.518	0.754	0.125	0.869	19.585	0.997	32.881	0.301	0.648	0.033
C4	0.196	4.415	0.756	0.116	0.883	19.113	0.998	31.435	0.309	0.649	0.031
P3	0.116	4.605	0.750	0.133	0.857	19.981	0.998	34.142	0.294	0.647	0.036
P4	0.149	4.435	0.744	0.199	0.868	19.238	0.997	32.295	0.301	0.633	0.032

Here, *R*^2^ is regression coefficient and *K*_0_, *K*_1_, kH, kKP and kHC are the release rate constants for zero-order, first-order, Korsmeyer–Peppas, Higuchi and Hixson–Crowell model, respectively.

**Table 3.** Acute oral toxicity studies of QSM-co-acrylamide hydrogels in mice

Sr. no.	Animal groups *n* = 2	Group I Control Mean ± SEM	Group II Treated (2 g/kg) Mean ± SEM
1	*Clinical observations*	Nil	Nil
2	*Body weight (g)*		
	Pretreatment	31.25 ± 2.1	29.6 ± 1.8
	After 1 day	33 ± 3.2	30.1 ± 1.6
	After 7 days	33.6 ± 2.4	29.8 ± 1.5
	After 14 days	32.5 ± 1.5	29.5 ± 1.4
3	*Water intake (mL)*		
	Pretreatment	16.25 ± 2.2	19.6 ± 1.6
	After 1 day	15 ± 3.0	20.1 ± 1.2
	After 7 days	18 ± 1.9	19.8 ± 1.7
	After 14 days	16.5 ± 1.7	18.5 ± 1.3
4	*Food intake (g)*		
	Pretreatment	3.6 ± 1.0	3.5 ± 0.8
	After 1 day	4.5 ± 0.2	4.1 ± 0.3
	After 7 days	3.5 ± 0.9	2.7 ± 0.6
	After 14 days	3.5 ± 0.7	2.5 ± 0.3
5	*Haematology*		
	Hb (g/dL)	14 ± 1.5	12 ± 1.5
	Total WBCs (10^3^/µL)	15.3 ± 0.16	13.9 ± 1.01
	RBC (10^6^/µL)	14.7 ± 1.3	15.1 ± 0.9
	Platelets (10^3^/µL)	889 ± 28.6	7564 ± 54
6	*Blood chemistry*		
	Liver profile		
	ALT	20.13 ± 1.4	19.79 ± 2.3
	Renal profile creatinine (mg/dL) (normal 0.2–0.9)	0.7 ± 0.6	0.5 ± 0.23
	Lipid profile S. cholesterol (normal 150–220 mg/dL)	156.9 ± 2.5	151.5 ± 2.4
	Serum triglyceride	79.15 ± 3.2	80 .77 ± 2.0
	*Histological examination*		
	Kidney	Normal	Normal
	Liver	Normal	Normal

**Table 4.** Effect of administering different 2 g/kg of QSM-co-acrylamide hydrogels on the organ weight of mice

Dose (mg/kg b.wt)	Heart	Lung	Liver	Kidney	Stomach
Control	0.43 ± 0.04	0.5 ± 0.02	5.15 ± 0.20	0.77 ± 0.09	1.59 ± 0.03
2 g/kg	0.40 ± 0.08	0.47 ± 0.02	5.10 ± 0.11	0.71 ± 0.07	1.54 ± 0.01

All weights are articulated as relative organ weight.

**Table 3.** Acute oral toxicity studies of QSM-co-acrylamide hydrogels in mice

Sr. no.	Animal groups *n* = 2	Group I Control Mean ± SEM	Group II Treated (1 g/kg) Mean ± SEM	Group III Treated (2 g/kg) Mean ± SEM
1	**Clinical observations**	Nil	Nil	Nil
2	**Body weight (g)**			
	Pretreatment	1130 ± 9	1160 ± 10	1152 ± 12
	After 1 day	1132 ± 6	1150 ± 14	1130 ± 20
	After 7 days	1135 ± 5.6	1150 ± 17	1145 ± 16
	After 14 days	1138 ± 12	1200 ± 12	1140 ± 6
3	**Water intake (mL)**			
	Pretreatment	126 ± 21	130 ± 13	132 ± 10
	After 1 day	128 ± 13	138 ± 12	129 ± 11
	After 7 days	130 ± 12	152 ± 16	139 ± 15
	After 14 days	125 ± 11	145 ± 13	152 ± 12
4	**Food intake (g)**			
	Pretreatment	253 ± 12	275 ± 11	250 ± 12
	After 1 day	240 ± 21	235 ± 26	239 ± 18
	After 7 days	255 ± 17	245 ± 23	245 ± 23
	After 14 days	253 ± 12	260 ± 21	255 ± 12
5	**Haematology**			
	Hb (g/dl)	13.7 ± 1.3	13.1 ± 2	12.5 ± 3
	Total WBCs (103 /µl)	10 ± 0.5	9 ± 0.7	9.5 ± 1.2
	RBC (106 /µl)	5.6 ± 0.6	5.0 ± 0.4	6.8 ± 1.9
	Platelets (103 /µl)	149 ± 1.2	168 ± 1.3	202 ± 1.8
6	**Blood chemistry Liver profile**			
	AST (U/L)	67.5 ± 12.81	59.5 ± 10.52	66.75 ± 12.75
	ALT (U/L)	78 ± 2.14	59 ± 1.60	71.5 ± 2.68
	ALP (U/L)	124.31 ± 2.3	129.16 ± 3.5	109.29 ± 3.2
	Albumin (2.5–4) (g/dl)	2.7 ± 0.5	2.9 ± 0.3	3.1 ± 0.6
	**Renal profile**			
	Creatinine (mg/dl) (normal 0.8–1.8)	1.1 ± 0.6	1.0 ± 0.4	1.2 ± 0.3
	Urea (m g/L)	0.29 ± 0.07	0.45 ± 0.08	0.53 ± 0.10
	**Lipid profile**			
	Cholestrol (10–80) (mg/dl)	38.20 ± 6	41.43 ± 3	32.37 ± 2.5
	Serum Triglyceride (46–68) (mg/dl)	38 ± 1.1	54.23 ± 1.3	41.75 ± 2.3

### Percentage yield

The percentage yield was estimated as 10.1% for QSM. Ashraf*et al*. also reported a 9.3% yield of quince mucilage by hot water extraction.

## Discussion

Stable QSM-co-Aam-based hydrogel was formulated by crosslinked polymeric network. The hydrogels based on quince mucilage were brownish in colour when dry and translucent when exposed to appropriate media.

### Swelling studies

#### Effect of polymer concentration on swelling behaviour of QSM-co-AAm polymeric network

The effect of polymer concentration was studied on the swelling action of various formulations. In Figure 2(a), swelling ratios are shown. The swelling of hydrogel samples was measured as a function of the time before saturation by measuring the amount of water consumed by the material. Swelling ratios of quince mucilage-based hydrogels with increased polymer concentration decreased marginally from QSM-co-AAm at pH 1.2 from 8.16 to 9.01. Swelling levels for QSM-co-AAm were elevated from 8.44 to 9.15 at pH 7.4. Quince mucilage is a hydrophilic anionic polymer. Hydrogel was strongly hydrated as the polymer content was increased due to the ionization of ionizable groups and the production of repulsive forces. Hydrogel porosity was also enhanced by high polymer concentration, which is attributed to reduced crosslinking density that resulted in improved swelling. The creation of a concentrated solution averts escaping of bubbles, which eventually increased porosity [18].

#### Effect of monomer concentration on swelling behaviour of QSM-co-AAm polymeric network

In Figure 2(b), the swelling ratios of QSM-co-AAm hydrogels are shown. The swelling ratio of M-3 and M-4 at pH 1.2 decreased from 10.02 to 7.13 and 10.27 to 7.76 at pH 7.4. There was no substantial difference between the swelling ratio at low and high pH levels. This was attributed to acrylamide being nonionic in nature. The complex balance swelling ratio was correlated with ionic osmotic strength, cross-connected mass and water preference for hydrogels. Due to improved crosslinking density, swelling of CO-co-AAm hydrogels was decreased by rising hydrogel AAm content. The reduction in the number of hydrophilic groups was caused by the intermolecular bonding of hydrogen between acrylamide amide groups and polymer hydroxyl groups. This resulted in less hydrogel affinity for water. The decline in hydrogel AAm content resulted in increased swelling due to reduced crosslinking and increased hydrogel microporosity [19].

#### Effect of crosslinker concentration on swelling behaviour of QSM-co-AAm polymeric network

In order to analyse the impact of crosslinker concentration on the swelling activity of various formulations of CO-co-AAm, hydrogel formulations were prepared with differing crosslinker content. The QSM-co-AAm swelling scale, as seen in Figure 2(c). It was observed that dynamic equilibrium swelling was minimized by increasing the MBA molar content. At acidic pH, the swelling ratio for CO-co-AAm was reduced from 7.99 to 7.14. For CO-co-AAm, the fundamental pH swelling ratio of hydrogels was reduced from 8.84 to 7.53. Hydrogels’ swelling activity was highly reliant on the degree of crosslinking. Owing to the increased number of crosslink points in the polymeric enclosure, the crosslinker material crosslink, density and the polymeric framework stability were increased while the hydrogel porosity was decreased. A loose packaging with a higher hydrodynamic-free volume was a network at lower crosslinking density, so the chains were able to accommodate more solvent molecules, resulting in high swelling. This clearly showed that the content of the crosslinker had a substantial influence on the swelling actions [20].

### FTIR characterization of QSM-co-AAm polymeric network

Figure 3 reveals the quince mucilage, AAm, QSM-co-AAm, metoprolol tartrate and QSM-co-AAm (loaded) IR spectra. The absorption peak of quince mucilage was due to N–H stretch at 3402 cm^–1^, to C–H stretch at 2933 cm^−1^, to 1606 cm^−1^ for N–H bending, to 1409 cm^−1^ for C–H bending, to 1354 cm^−1^ for C–H bending vibration–CH_2_, to 1041 cm^−1^ for CH stretching [21].

Symmetrical and asymmetrical stretching of the N–H group leads to the presence of the band at 3327 cm^−1^ in the IR spectra of acrylamide. Vibration bands of amide and acid groups were detected due to characteristic C=O stretching at 1666 cm^−1^ and 1913 cm^−1^ and a peak of 1606 cm^−1^ for the CH=CH_2_ group [22]. The characteristic peaks at 3929.00 cm^−1^ are due to O–H in the IR spectrum of the pure drug metoprolol tartrate, at 1595.33 cm^−1^ attributable to C=O and at 1242.09 cm^−1^ referring to the C–N party [23].

A peak observed at 1676 cm^−1^ was the carbonyl group of amide movement of the grafted acrylamide chain in IR spectra of CO-co-AAm, which was not observed in quince mucilage, suggesting AAm grafting on *Cydoniaoblonga*. When the loaded hydrogel IR spectra were compared with the metoprolol tartrate IR spectra, it was reported that owing to the O–H, C=O and C–N groups, characteristic drug peaks were present at 3784 cm^−1^, 1608 cm^–1^and 1257 cm^–1,^ respectively. Peaks at 2866 cm^–1^ and 1116 cm^–1^ were attributed respectively to the linkage of the C–H group and alkyl aryl ether. It was verified that there was no interaction between drug and other hydrogel contents.

### SEM of freeze-dried QSM-co-AAm polymeric network

As shown in Figure 4, the surface morphology of QSM-co-AAm was studied by SEM. It was evident that hydrogel shows a microporous surface, with heterogeneously spaced large and irregular pores. Such anatomy facilitates the absorption of high concentrations of liquid by hydrogel [24].

### In vitro drug release from QSM-co-AAm polymeric networks with different polymer concentration

Figure 5(a) indicates the percentage of drug release of QSM-co-AAm hydrogels with different polymer concentrations. The drug release percentage for QSM-co-AAm increased from 84.19 to 86.97 at pH 1.2, and the drug release percentage for QSM-co-AAm hydrogels increased from 84.97 to 87.19 at baseline pH 7.4. This reported rise in release was due to more hydrophilic groups being present. Hydrogel becomes highly hydrated by increasing the polymer content, leading to the presence of further hydroxyl groups, which in turn contributes to improved drug release [25].

### In vitro drug release from QSM-co-AAm polymeric networks with varying concentration of acrylamide

Figure 5(b) indicates the percentage of drug release of QSM-co-AAm hydrogels with different amounts of monomer. The drug release from M-3 and M-4 at acidic pH was reduced from 98.02 to 85.54, and basic pH (7.4 pH) was reduced from 98.27 to 85.76, respectively. Due to the nonionic nature of acrylamide, drug release was not based on the pH of the medium. Hydrogel crosslinking density was improved by increasing the acrylamide content and microporosity was reduced. Owing to the use of the hydrophilic group in hydrogen bonding between the hydrophilic groups of quince mucilage and the amide group of acrylamide, hydrophilicity was also diminished, resulting in decreased swelling and less drug release percentage [26].

### In vitro drug release from QSM-co-AAm polymeric networks with varying crosslinker concentrations

Figure 5(c) indicates the percentage of drug release of QSM-co-AAm hydrogels with different amounts of crosslinker. The drug release percentage was lowered from 86.01 to 84.75 for CO-co-AAm hydrogels. It decreased from 86.19 to 84.86 for QSM-co-AAm hydrogels at high pH. The crosslinking density was greater due to higher crosslinker concentration, and the hydrogel shape was stern. More hydroxyl groups have been consumed in crosslinking reactions due to the generation of more crosslink points. As a result, the space of the network is reduced, and less water enters the hydrogel. For mechanical stability, higher MBA concentration was advantageous, but the lower porosity and hydrophilicity resulted in a decreased rate of drug release at the same time [27].

### Drug release kinetics

The pattern of drug release in buffer solutions of pH 1.2 and pH 7.4 was studied. The data collected at pH 7.4 were put in Korsmeyer–Peppas, Higuchi and Hixson Crowell zero-order, first-order models for the assessment of the pattern of drug release. On the basis of the best fitness of the release model, the most effective release process was elucidated. The release models were estimated by taking regression value 1.

Regression coefficient values for all models of drug-loaded hydrogels with separate monomer, crosslinker and polymer contents are shown in Table 1, 2. The best-fit model for all formulations was found to be Korsmeyer–Peppas based on the maximum regression coefficient value (*R*^2^). The value of *n* for hydrogel QSM-co-AAm observed was less than 0.45. Fickian was the drug release mechanism for QSM-co-AAm based on *n* value [28].

### Acute oral toxicity of QSM-co-AAm polymeric network

Acute oral toxicity provides estimation of toxic doses and also gives information about therapeutic index (LD50/ED50) [29]. No mortality of treatment was observed in mice and rabbits. During 14 days treatment time, no toxic effects were witnessed. Quince mucilage polymeric network-treated rabbits and mice physical examination showed no toxic signs like alteration in skin, eyes, mucosal membrane, tremors, diarrhoea, salivation, behavioural pattern, sleep order, faecal pattern and coma. No mortality was observed even at highest doses of quince extract hydrogels [30].

### Body weight, food and water consumption

The body weight, food and water consumption of treatment and controlled groups are shown in Table 3. Minor gradual decrease in weight was observed in the treatment group of mice. There was no significant difference in weight, food and water consumption of treatment group (hydrogel powder intake) when compared with control (placebo) on completion of 14 days. A gradual decrease followed by increase in weights of treatment groups was observed, which was not significantly different when compared with the control group. Food and water intake in the treated group of rabbits was not considerably different from the control group (Table 5).

### Haematology and blood chemistry

Blood chemistry and haematological parameters of mice and rabbits are tabulated in Tables 3 and 4, respectively. Haematological constituents are expedient in the checking of specific dosage form toxicity. The investigation of blood parameters is the principle of founding the state of health of an animal and thus influencing the effect of the drug delivery system components on the blood composition. Blood testing can therefore be deliberated as a suitable impression of long-term dosage form status. Changes in haematological and biochemical parameters in animals entitle their normal physiological state. The present study depicts that biochemical results expose no substantial alteration with respect to the control. The lack of a vital dissimilarity in liver enzymes level displays the protective hepatotoxic state, and absence of significant differences in creatinine and urea also demonstrate the nephroprotective property of QSM-co-AAm hydrogels on the health of rabbits. The complete blood, renal, liver and lipid profiles were similar to the control group and appeared in normal range, which demonstrated safety profile of QSM-co-AAm hydrogels [31].

### Gross necropsy

Histopathological study confirmed nontoxicity of QSM-co-AAm because no change was observed in organs. The relative organ weight/100 g (body weight) did not show substantial difference when compared with control group as tabulated in Table 4. Upon histological evaluation of kidney and liver, no inflammation and degeneration was observed. Lack of lesions in histopathological examination suggested QSM-co-AAm hydrogels’ safety in animals. Hence, polymeric network did not turn out to be toxic for these organs, thereby signifying the safety of the polymeric network. Convincingly, no gross variance in histopathological observation was found between the control and treatment groups similar to haematological and biochemical biomarkers, attributed to the normal functioning of vital organs. The consequences of acute oral toxicity evaluation accessible showed that there was no toxic reaction or histopathological vagaries prompted after maximum dose level of QSM-co-AAm polymeric network. Thus, this drug delivery system might be a nontoxic applicant for use in the biomedical field, particularly in oral drug delivery system [32,33].

### Conclusion

The concept of formulating chemically crosslinked polymeric network based on QSM offers a proper, functional approach to accomplish an enduring therapeutic consequence by continuously releasing the drug over extended period of time. Free radical polymerization is a conspicuous and more cost-effective method to impart useful functionalities to drug delivery system, hence this research work will be dividend to sustained drug delivery system. Toxicity study’s findings show that the developed polymeric network is nontoxic and safe and may emerge in the future as a better choice for the delivery of oral antihypertensive drugs.
